# Comparative studies between mice molars and incisors are required to draw an overview of enamel structural complexity

**DOI:** 10.3389/fphys.2014.00359

**Published:** 2014-09-19

**Authors:** Michel Goldberg, O. Kellermann, S. Dimitrova-Nakov, Y. Harichane, A. Baudry

**Affiliations:** ^1^INSERM UMR-S 1124, Cellules Souches, Signalisation et PrionsParis, France; ^2^Université Paris Descartes, Sorbonne Paris Cité, UMR-S 1124Paris, France

**Keywords:** incisor, molar, serotonin receptor, Hunter-Schreger bands, maturation, gene deletion

## Abstract

In the field of dentistry, the murine incisor has long been considered as an outstanding model to study amelogenesis. However, it clearly appears that enamel from wild type mouse incisors and molars presents several structural differences. In incisor, exclusively radial enamel is observed. In molars, enamel displays a high level of complexity since the inner part is lamellar whereas the outer enamel shows radial and tangential structures. Recently, the serotonin 2B receptor (5-HT_2B_R) was shown to be involved in ameloblast function and enamel mineralization. The incisors from 5HT_2B_R knockout (KO) mice exhibit mineralization defects mostly in the outer maturation zone and porous matrix network in the inner zone. In the molars, the mutation affects both secretory and maturation stages of amelogenesis since pronounced alterations concern overall enamel structures. Molars from 5HT_2B_R KO mice display reduction in enamel thickness, alterations of inner enamel architecture including defects in Hunter-Schreger Bands arrangements, and altered maturation of the outer radial enamel. Differences of enamel structure were also observed between incisor and molar from other KO mice depleted for genes encoding enamel extracellular matrix proteins. Thus, upon mutation, enamel analysis based exclusively on incisor defects would be biased. In view of the functional relationship between enamel structure and tooth morphogenesis, identification of molecular actors involved in amelogenesis requires comparative studies between mice molars and incisors.

## Introduction

In humans, two renewals of the dentition through life occur corresponding to diphyodonty (Whitlock and Richman, 2013). Successive deciduous and permanent sets of teeth with limited growth are formed. The presence of tooth bud adjacent to the deciduous teeth allows building the permanent teeth. Thus, postnatal tooth formation occurs, implying that enamel organ participates to the crown formation of the nascent teeth. Although the mechanisms underlying enamel formation during embryogenesis have been partly elucidated (Laugel-Haushalter et al., 2013), almost nothing is known concerning the signals promoting enamel bud differentiation of the permanent teeth. The thickness and the mechanical properties of permanent vs, deciduous tooth differ indicating that the enamel mineralized microstructures built by ameloblasts depends on tooth identity. Conversely, monophyodont teeth corresponding to one single tooth generation characterizes rodents. Thus, the use of rodent models is dealing with deciduous teeth. We remind that in rodent, molars have limited growth whereas incisors are continuously growing teeth. The rodent represents an exception since ameloblasts do not disappear throughout life ensuring permanent formation of enamel on the labial surface of incisor.

Enamel covers dentin and dental pulp in the crown of the tooth. It is the hardest tissue in the body due to the high mineral content (96%) and a very little protein level (less than 1% organic material). The root is recovered by cementum that participates to the tooth attachment to bone. Dentin and dental pulp derive from neural crest while enamel originates from neuroectoderm. Enamel is the only epithelial derived calcified tissue in vertebrates. This avascular tissue is formed during tooth development by ameloblasts, which are lost after deciduous or permanent tooth eruption.

In mammals, enamel structures display different levels of complexity (Koenigswald and Clemens, 1992). The assembly of hydroxyapatite monocrystals along the so-called Burgers vectors and circuit leads to the formation of individual crystallites (Arends and Jongebloed, 1979). Bundles of crystallites are packed in rods or interrod enamel. Rods contribute to the three-dimensional organization of enamel, either as linear structures, or forming sophisticated spatial constructions characterized as Hunter-Schreger (HS) bands (Osborn, 1990; Risnes, 1990).

## Enamel structural differences between incisors and molars from wild type (WT) mouse

In rodent, three-dimensional arrangement of enamel types, named schmelzmuster, are well characterized. The C-type schmelzmuster of molar is related to a basal ring of lamellar enamel. This C-type is not present in the incisor. The P-type corresponds to radial enamel found in the outer area of molars and predominant in the incisors. The S-type formed by thick HS bands is mostly identified in molars (Koenigswald, 2004).

In rats and mice, the occurrence of a large diastema between the incisor and molar is at the origin of the absence of canine and premolars. A stem cell niche associated with the basal lamina is present in molar and is very active in growing incisor. The absence of stem cells has been reported in diastema area (Lesot and Brook, 2009). The crown of rodent molar is protected by enamel while the root, as in human, is covered by cementum. Of note, in rodent incisor, the presence of enamel restricted to the labial side fulfills biomechanical requirement. The newly formed enamel slides in the bony socket. The lingual root-like part including both the cementum and alveolo-dental ligament (Steinfort et al., 1989) ensures the insertion of the continuous erupted incisor.

**Figure 1:**
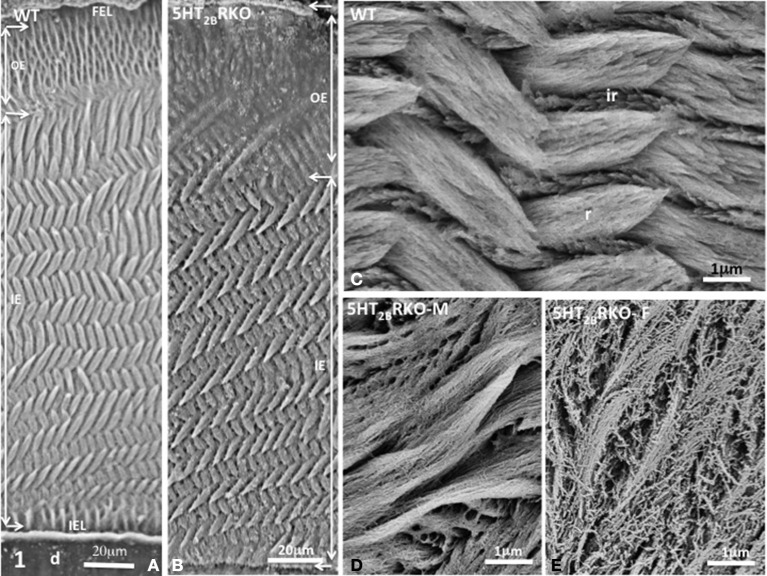
**(A)** Transverse section of Wild Type (WT) incisor. D, dentin; IEL, inner enamel layer; IE, inner enamel; OE, outer enamel; FEL, final enamel layer. The lamellar pattern is mostly detectable in the inner enamel. **(B)** Transverse section of the incisor of 5HT_2B_R KO mice. The outer enamel (OE) is more resistant to acid etching than the inner enamel (IE), suggesting residual accumulation of the extracellular matrix. **(C)** In the WT mouse, crystallites are tightly packed inside rods (r) or in interrod enamel (ir). **(D)** In the male mutant mouse (5HT_2B_R KO-M), porosities appear in rods and interrods where crystallites are less densely packed. **(E)** In the female KO (5HT_2B_R KO-F) the porous appearance is increased. Intercrystallite spaces are enlarged.

Most laboratories have worked on rodent incisor since they exploited the dynamics of enamel formation of the continuously growing incisor (Leblond and Warshawsky, 1979). The advantage of the incisor model is that all the spatio-temporal steps occurring during amelogenesis are clearly identified. Initiation of enamel formation is recognized in the apical zone called the zone of pre-secretion where polarized ameloblasts are facing the pulp. Then, dentin starts to be formed. Signals exchanges between odontoblasts and ameloblasts occur and epithelio-mesenchymal interactions ensure the differentiation of ameloblast in tooth secretory cells. Enamel extracellular matrix proteins are secreted forming a network for mineral deposition. Radial rods and interrod enamel are formed first in the inner enamel and then in the outer enamel concomitant with the backward migration of ameloblasts. Mineralization occurs at the border between the translucent zone and the chalky enamel, a zone where both hydroxyapatite deposition and crystal nucleation occur. The extensive mineralization of enamel involves the post-secretory step. Ameloblasts that are forming a thin layer of outer enamel secrete proteases such as the metalloprotease MMP20 and kallikrein 4 (KLK4) (Smith et al., 2011) which degrade the organic matrix and reduce ECM content from 20 to 0.4–0.6% (in weight). As mineral crystals grow most of the cleaved peptides are removed. This leads to final enamel maturation that spatially takes place prior eruption. In rodents, the grinding activity leads to incisor abrasion that is compensated by continuous tooth eruption. In the incisor, enamel displays a uniserial lamellar pattern of prisms in the inner enamel, whereas radial prisms are parallel in the outer enamel (Moinichen et al., 1996) (Figure 1A). Tightly packed bundles of crystallites form rods and interrod enamel (Figure 1C).

**Figure 2:**
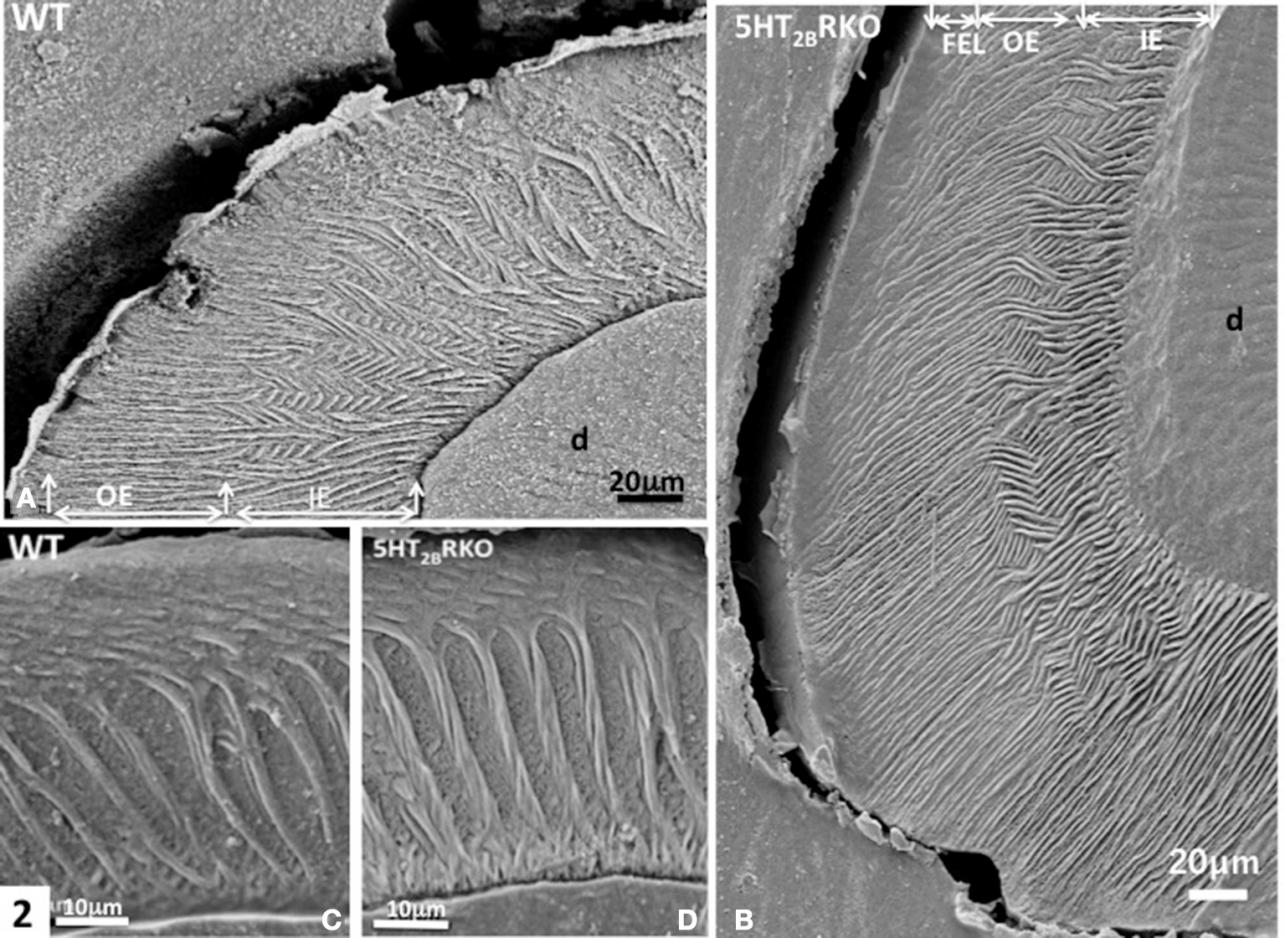
**(A)** WT molar. In the inner enamel (IE), a feather-like structure is observed, whereas in the outer enamel (OE) radial rods are perpendicular to the enamel surface. D, dentin. **(B)** In the molar of 5HT_2B_R KO mice, rod bending and decussation are seen in the inner enamel (IE), whereas in the outer enamel (OE) radial rods are parallel and perpendicular to the outer final enamel layer (FEL) The Hunter-Schreger Bands are barely detectable. **(C)** WT enamel molar. Hunter-Schreger bands are located in the inner enamel, whereas oblique and radial rods are mostly detected in the outer enamel. **(D)** In the 5HT_2B_R KO mice, twisted and thicker rods contribute to the formation of Hunter-Schreger Bands, which are located in the inner enamel. Along the dentino-enamel junction a thin layer of aprismatic enamel is detected.

**Figure 3:**
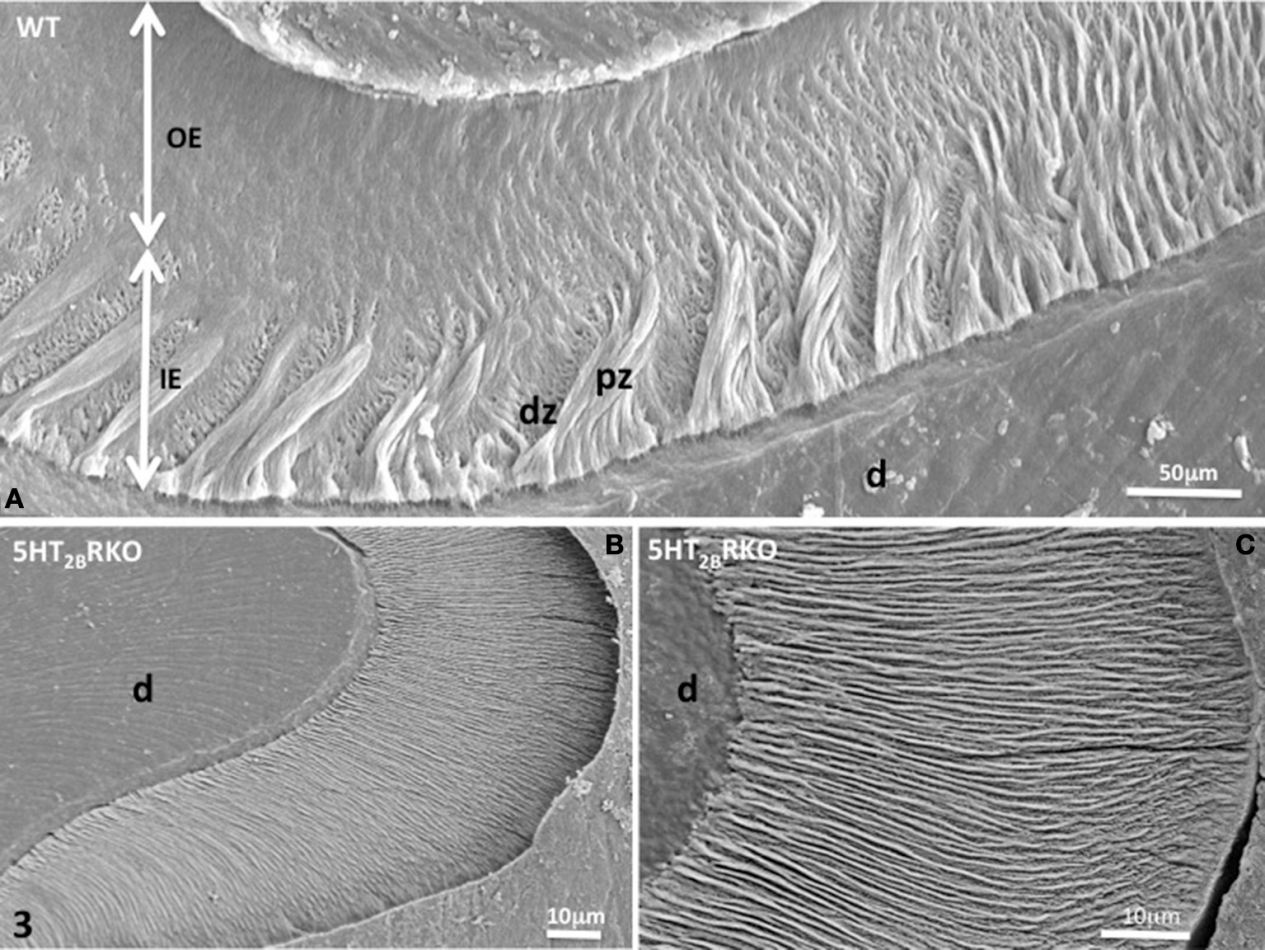
**(A)** WT molar enamel. In a grove between two cusps, large diazones (dz) and parazones (pz) occupy the inner enamel (IE) near the dentino-enamel junction. Radial rods are located in the outer enamel (OE). **(B)** 5HT_2B_R KO molar rod enamel is continuous and has a radial appearance. **(C)** Radial enamel at the tip of a cusp. d, dentin.

**Figure 4:**
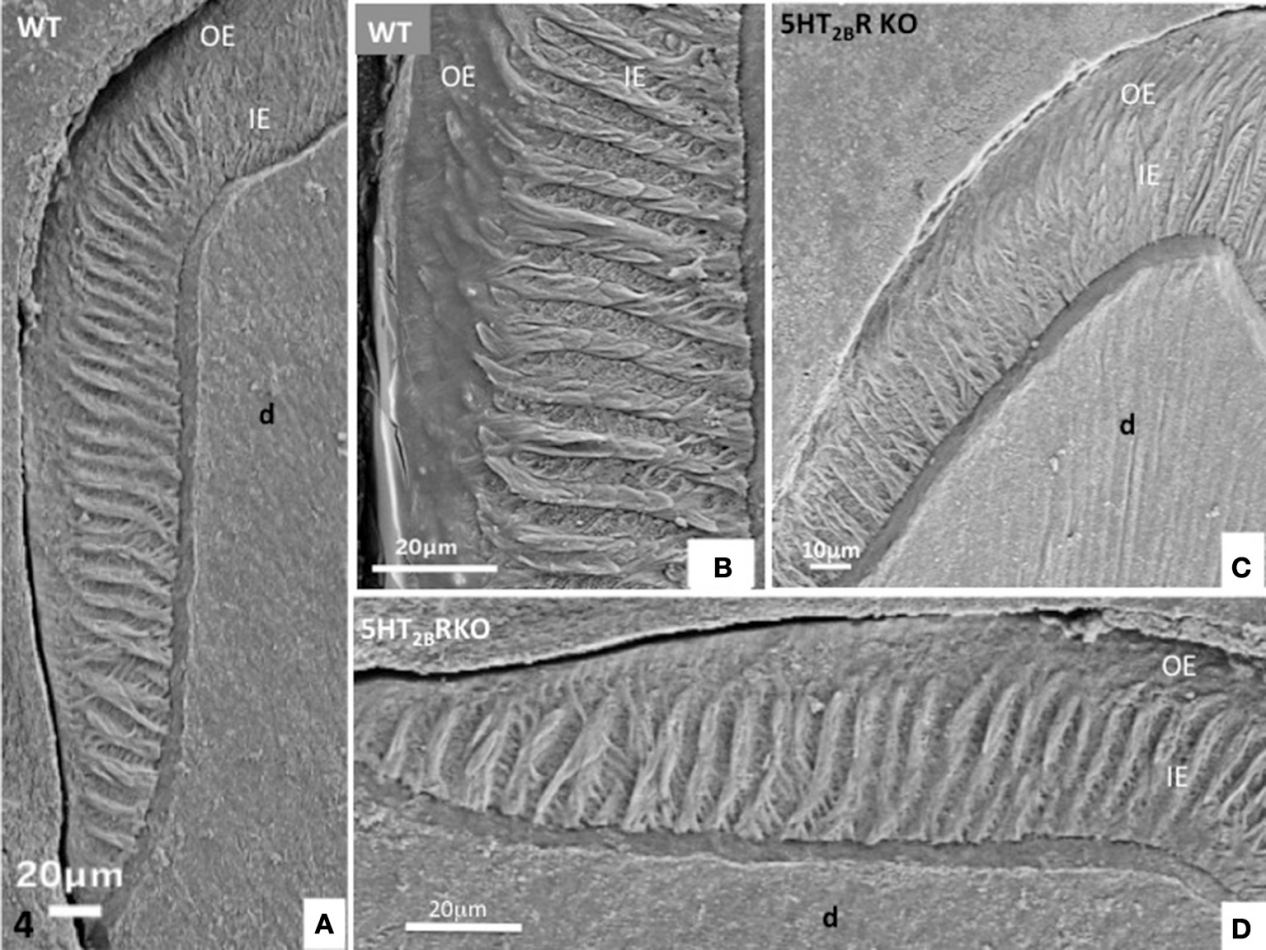
**(A,B)** Lateral aspect of WT enamel molar. Hunter-Schreger bands occupy the inner enamel (IE) whereas the outer enamel (OE) displays an amorphous appearance. **(C,D)** In the molar of 5HT_2B_R KO mice, alternatively diazones and parazones occupy two third of the inner enamel which looks more porous compared to WT.

In rodents, the enamel of molar displays a multifaceted rod pattern with more variable structures than in the incisor. In the molar, four characteristics are noted: (1) decussation of rods observed in the inner third and absent in the upper ridges and in the bottom of grooves, (2) a feather-like rod organization also forms lamellar enamel in the inner third (Figure 2A), (3) all rods seen in the outer enamel are radial [right angle to the surface (Figures 2A,C)] and/or exhibit the same oblique orientation tangential to enamel surface (Figure 2C) (Koenigswald and Clemens, 1992), (4) incremental lines with a regular periodicity constitute the superficial aprismatic enamel (Lyngstadaas et al., 1998). Enamel organization is strongly dependent on the zone investigated (Risnes, 1979). A strong link may be established between regional variations of enamel structure and the rod-interrod scaffold, which allows strain resistance (Figures 2–**4**). The predominant structure consists in several waves of rod decussation in the inner enamel and in rods which are radial in outer enamel (Lyngstadaas et al., 1998) (Figure 2A).

In addition, HS bands increase the architectural complexity. Two to three rods form a helix due to the cervical translocation of some of the rods in the transverse plane of the tooth (Osborn, 1990). HS bands result from anti-parallel orientation of diazones (dz) (transversely cut rods) and parazones (pz) (longitudinally cut rods) in the lamellar inner enamel (IE) (Figure 2C). Enamel architecture is based on the continuity of spirally arranged rows at least in the inner third of dental enamel (Hirota, 1982). The double-coiled helix tends to enlarge gradually the volume of enamel, leading to its thickening by helicoidal growth. HS bands represent a plywood structure that further enhances the axial compression strength below the cusp (Figure 3A). HS bands visualized in the lateral aspects of molar (Figures 4A,B) also contribute to lateral resistance. HS bands are undetectable in the outer enamel and instead paralleled radial rods are present in the deep part of occlusal groves (Figures 3B,C).

These differences in enamel organization between mice incisor and molars imply that the genetic intrinsic program governing ameloblast differentiation involves distinct mechanisms during embryogenesis (molar) vs. post-natal life (incisor). Generation of knockout mice displaying differential defects between incisors and molars may shed light on new actors implicated in amelogenesis.

## Differential structural enamel alterations between incisors and molars in 5HT_2B_R knockout mice

Recently, the serotonin 2B receptor (5-HT_2B_R) was shown to be involved in ameloblast function and enamel mineralization. We may remind that during embryogenesis, serotonin (5-HT) contributes to the differentiation of neuroectodermal, neural crest, and mesodermal derivatives (Moiseiwitsch and Lauder, 1995; Gaspar et al., 2003). In addition, 5-HT_2B_R is present in the first branchial arch where tooth buds form (Choi et al., 1997; Lauder et al., 2000). In adult, this receptor controls tissue non-specific alkaline phosphatase (TNAP) activity (Baudry et al., 2010), and thereby bone mineralization (Collet et al., 2008). Inactivation of the 5HT_2B_R [5HT_2B_R knockout (KO) mice] disturbs enamel formation (Harichane et al., 2011; Dimitrova-Nakov et al., 2014).

In 5HT_2B_R KO incisor, the outer enamel (OE) is thicker than in WT and has an amorphous appearance (Figure 1B) revealing anomalies in post-secretory ameloblast functions. We used a 1.3% nitric acid treatment for three periods of 5 s (Risnes, 1990) that permits limited demineralization at the surface section thus allowing to still visualize mineral structures. The inner enamel (IE) of 5HT_2B_R KO mice displays a series of decussation more accentuated than in WT with minor but significant changes in orientation of rods (r) and interrods (ir) (Figures 1B,C). At higher magnification, porosities are observed in the enamel structure of 5HT_2B_R KO mice with a sexual dimorphism. In 5HT_2B_R KO male, the crystallites are thinner and less densely packed (Figure 1D), whereas in 5HT_2B_R KO female (Figure 1E), the porosities are more pronounced with unpacked crystallites, and swollen inter crystallites void spaces. Such sexual dimorphism was also noted for bone defects in 5HT_2B_R KO mice (Collet et al., 2008).

In molar, 5HT_2B_R depletion affects overall enamel structures. The thickness of enamel in 5HT_2B_R KO mice is decreased possibly due to the observed defects of enamel organization/mineralization and/or the increase in occlusal surface abrasion in enamel-free areas. Interestingly, HS bands almost disappear in the upper part of the cusp. Conversely, HS bands are thicker in the proximal parts of the teeth involving the self-association of additional rods, exhibiting a twisted appearance (Figures 4C,D). Near the tip of the cusp, the feather-like structure normally formed by rods and inter-rods is no more observed. Instead, after a series of bendings in the inner enamel, rods and interrod enamel follow radial orientation in the outer enamel (Figure 2B). These results indicate that the absence of 5HT_2B_R alters the withdrawal and the wavy route of secretory ameloblasts.

Near the crests of the cusps, closest to the enamel surface, the post-secretory functions of maturing ameloblasts contribute to the formation of a final enamel layer, which is aprismatic, and resulting from ECM re-internalization (Figures 3B,C, 4C,D).

Nearby the top of the cusps, rods and interrod enamel follow radial direction, without noticeable difference between the inner and outer enamel (Figures 3B,C).

In the proximal enamel extending from the cervical zone to the top of the cusp, the inner enamel (IE) is formed alternatively by thick parazones and thinner diazones forming a plywood structure, a three-dimensional scaffold allowing better resistance to axial pressures excerpted on the crown (Figures 4C,D). The outer enamel is formed by parallel alignment of rods, and covered by a thin aprismatic outer layer (OE) (Figures 4A,B). A similar architecture of enamel structures is seen in matched areas of WT and 5HT_2B_R KO molars, but at higher magnification the whole enamel appears to be more porous in absence of 5HT_2B_R (Figures 4C,D).

## Lessons from incisors and molars structural analysis in knockout mouse models

Mice KO for genes encoding proteins known to be present in enamel matrix all exhibit alterations in molar and incisor enamel as compared to WT. In the case of periostin, postnatal defects in incisors and molars predominantly concern dentin (enormous increase at 5 months). Minor alterations are observed in enamel probably resulting from impaired mechanical response (Ma et al., 2011). For ameloblastin and enamelin, enamel of incisor and molar are extremely thin and irregular with a rough surface (Smith et al., 2009; Chun et al., 2010).

Amelogenin is the most abundant enamel matrix protein (90%) and is essential for amelogenesis. Amelogenin-deficient mice display an amelogenesis imperfecta phenotype (Gibson et al., 2001). The amelogenin KO incisors appear as abnormal teeth with chalky-white discoloration, longitudinal furrows, and broken tips. At an ultrastructural level, the characteristic prism pattern is completely absent. The amelogenin KO molar cusps are abraded and the disorganized hypoplastic aprismatic enamel display reduced thickness.

Enamel matrix proteinases suck as KLK4 and MMP20 are secreted in tooth enamel matrix during the maturation stage of amelogenesis. They have a role in the degradation of matrix proteins to promote the export of the cleaved forms from the hardening enamel. In mice lacking KLK4, the incisor enamel has a normal thickness and no change in its overall organization in terms of rod/inter-rod structure. The developmental defect in the enamel rods is restricted to the first formed inner enamel near the dentino-enamel junction. Thinner and structurally abnormal incisor enamel is observed in MMP20 KO mice. As concern molar, both KLK4^−/−^ and MMP20^−/−^ display occlusal attrition (Smith et al., 2011).

The Na^+^-independent anion exchanger 2 (Ae2) is associated to the basolateral membrane of ameloblasts and is involved in the pH regulation during the maturation phase of amelogenesis. Ae2 maintains low pH in enamel layer in order to catalyze proteins hydrolysis and/or to drive the mineralization process. In Ae2 KO mice, the decrease in mineral content and the presence of high organic matrix level in incisors and molars suggest that in the absence of Ae2, mineral ion transport as well as protein resorption are impaired. These changes are more pronounced in incisors, which in addition, lacks the typical yellow-orange enamel pigmentation normally observed in the WT incisor. Consistent with the loss of mineral, Ae2^−/−^ erupted molars exhibit severe abrasion and the occlusal surface is almost devoid of enamel (Lyaruu et al., 2008).

Ameloblasts express αvβ 6 integrins which (i) mediate cell matrix adhesion and thereby may have a role in the backward migration of ameloblasts in molars or cell translation in incisors, (ii) trigger signaling pathways that regulate the cell cycle, shape and motility as well as the mechanical status of extracellular matrix. In Itgb6^−/−^ incisors, the enamel prism organization was completely lost. Further, incisors are abnormally white in absence of αvβ 6 integrins. Itgb6^−/−^ molar have also hypomineralized enamel as inferred by extensive wear at their occlusal surfaces and much faster rate of attrition compared to WT molars (Mohazab et al., 2013).

## Conclusion

Although enamel mineral composition does not vary much between incisors and molars, enamel microstructural complexity in term of three-dimensional organization of rods and interrods is specific of each tooth type. This well organization structure of enamel is though to be controlled by enamel matrix proteins and proteolytic enzymes whose depletion promotes different degree of enamel hypoplasia. Of note, in all the KO mice described here, enamel is still formed. The more severe phenotype is observed in amelogenin KO mice that mimicks the human amelogenesis imperfecta. As described above, defects related to genetic depletion may have differential effects on molar vs. incisor. They mostly affect the optimal hydroxyapatite crystal growth in the enamel maturation zone and not during the initial forming steps of amelogenesis.

### Conflict of interest statement

The authors declare that the research was conducted in the absence of any commercial or financial relationships that could be construed as a potential conflict of interest.
